# Photocatalytic degradation of industrial acrylonitrile wastewater by F–S–Bi–TiO_2_ catalyst of ultrafine nanoparticles dispersed with SiO_2_ under natural sunlight

**DOI:** 10.1038/s41598-020-69012-z

**Published:** 2020-07-23

**Authors:** Feng Ouyang, Hanliang Li, Zhengya Gong, Dandan Pang, Lu Qiu, Yun Wang, Fangwei Dai, Gang Cao, Bandna Bharti

**Affiliations:** 1 State Key Laboratory of Urban Water Resource and Environment, Harbin Institute of Technology, Harbin, 150090 People’s Republic of China; 2 School of Civil and Environmental Engineering, Harbin Institute of Technology, Shenzhen, 518055 People’s Republic of China; 3 Henan University of Urban Construction, Pingdingshan, 467036 People’s Republic of China; 4 Tonson Tech Automation Equipment CO., Ltd, Shenzhen, 518100 People’s Republic of China; 5 Shenzhen Key Laboratory of Organic Pollution Prevention and Control, Harbin Institute of Technology, Harbin, People’s Republic of China

**Keywords:** Nanoscale materials, Pollution remediation

## Abstract

Highly active photocatalyst, having certain anti-ionic interfering function, of F, S and Bi doped TiO_2_/SiO_2_ was used for the first time to degrade the organic pollutants in acrylonitrile industrial wastewater under natural sunlight. The photocatalyst were prepared and characterized by UV–Vis, XRD, TEM, EDS, Nitrogen physical adsorption and XPS technique. UV–Vis analysis revealed addition of F, S and Bi into the lattice of TiO_2_ led to the expansion of TiO_2_ response in the visible region and hence the efficient separation of charge carrier. The photocatalytic potential of as prepared catalyst to degrade acrylonitrile wastewater under simulated and natural sunlight irradiation was investigated. The extent of degradation of acrylonitrile wastewater was evaluated by chemical oxygen demand (COD_Cr_). COD_Cr_ in wastewater decreased from 88.36 to 7.20 mgL^−1^ via 14 h irradiation of simulated sunlight and achieved regulation discharge by 6 h under natural sunlight, illuminating our photocatalyst effectiveness for refractory industrial wastewater treatment. From TEM results, we found that SiO_2_ could disperse the photocatalyst with different component distributions between the surface and the bulk phase that should also be responsible for the light absorption and excellent photocatalytic performance. The XPS analysis confirmed the presence of surface hydroxyl group, oxygen vacancies.

## Introduction

Acrylonitrile is considered as a significant industrial chemical, originated by the direct oxidation of propylene with ammonia. It is extensively used for the preparation of synthetic rubber and resin, plastic and acrylic fiber^1,2^. Various types of organic pollutants are formed during the production of acrylonitrile^3,4^ which has definitely induced serious impact on environmental and public health. Owing to its low bioavailability, high toxicity and mingled composition, acrylonitrile production wastewater has been directed as one type of refractory organic wastewater^5^. Therefore, it is necessary to develop a safe and efficient technology for the treatment of acrylonitrile wastewater. Various methods have been reported for the treatment of acrylonitrile wastewater, among those methods photocatalysis acquired much attention over the past decade. Since, photocatalytic reaction under sunlight irradiation is more energy-advantageous, and a lot of researchers have made vast efforts to realize the industrialization of photocatalytic treatment of industrial wastewater under sunlight^6–8^. However, there were few successful reports under sunlight because of the complexity of industrial wastewater^9–11^. Thus, photocatalytic treatment of industrial wastewater under sunlight was a great challenge for the researchers. Watanabe^12^ have reported that photocatalysis would cause a new environment revolution twenty years ago.

It is well known that, semiconductor-based photocatalysts have been investigated as an auspicious material for the solar energy conversion in regard to the breakdown of hazardous organic pollutants^13^. Across various photocatalysts, titanium dioxide (TiO_2_) is known as the most determined material because of its chemical stability, high oxidation potential, nontoxicity and physical stability^14^. However, the use of TiO_2_ in photocatalysis are limited because of its certain drawbacks like: their large band gap^15^, which means that in solar energy processes, only UV light can be utilized and their low photocatalytic efficiency because of the fast recombination rate of electron–hole pairs. Therefore, many efforts have been promoted to reduce the bandgap of TiO_2_ by doping or by band gap engineering^12–19^. In our previous study, the high photocatalytic activity of F-doped TiO_2_ was attributed to the increase in the number and strength of surface acid sites^20^. It was explained that F-doping led to the creation of surface oxygen vacancies^17^, or the increase of Ti^3+^ state^21^. On the other hand, higher photocatalytic activity of S doped sample was attributed to the increase in the surface Bronsted and Lewis acid sites^22^. Samantaray indicated that sulphate radical impregnation decreases the crystallite size and stabilized the anatase phase of TiO_2_^23^. In addition, Bi doped TiO_2_ exhibited a red shift in the optical adsorption and Bi^3+δ+^ species played a vital role in minimizing the electron hole recombination^16^. According to Li et al.^24^, Bi doping into TiO_2_ generates a new intermediate energy level below the conduction band edge of TiO_2_, extending the absorption in the visible region and enhanced their photocatalytic efficiency. On the other hand, SiO_2_ was used usually as a supporter, and its dispersing effect on nanoparticle size as well as that with oxidativity has not been reported.

To improve the photocatalytic activity of TiO_2_ for the decomposition of organic pollutants in acrylonitrile wastewater under solar light irradiation, we modified TiO_2_ with the combination of silica and F, S, Bi doping (F–S–Bi–TiO_2_/SiO_2_). The dispersion of SiO_2_ produced ultrafine nanoparticles. We have found that silica dispersion changed the aggregation state, constituent distribution in amount and morphology of the nanocatalyst, which was responsible for light absorption and increased photocatalytic activity^20^. We first degraded the acrylonitrile simulated wastewater and then degraded the acrylonitrile wastewater. This photocatalyst exhibited excellent performances in both the photocatalytic decompositions of organic pollutants under simulated and natural sunlight. So, our approach is an important attempt for the photocatalytic treatment of industrial wastewater.

## Characterization

The valence states on the surface of catalysts were analyzed by a Thermo ESCALAB 250XI X-ray photoelectron spectrometer (America) using Al Kα (hn = 1,486.6 eV) as a radiation source. The irradiation of simulated sunlight and natural sunlight intensity was measured with a FZ-A RADIOMETER irradiance meter (China). From 6 am–8 pm, it was measured at a certain interval as shown in Table S1. Tecnai G^2^ F30 TEM was used to analyze the physical structural characteristics of the photocatalysts. The samples were ultrasonically dispersed in ethanol. The suspension was deposited on a Lacey-carbon film, which was supported on a copper grid. The particle size distributions of catalysts with or without SiO_2_ dispersant were calculated with NIH software using TEM image treatment. The crystalline phases of the photocatalysts were determined by X-ray diffractometer (RIGAKU, D/Max 2500PC, Japan) at a scanning rate of 6° min^−1^ in the 2θ angle range of 10°–80° using Cu Kα radiation combined with nickel filter. The accelerating voltage and the applied current were 40 kV and 200 mA, respectively. Crystallite sizes were calculated according to Scherrer equation:

1 $$ L = K\lambda /B\cos \theta ,\quad B^{2} = B_{mea}^{2} - b_{ins}^{2} $$

where L, K, λ and θ are the average crystal size, the shape factor for spherical crystallites, the X-ray wavelength and Bragg diffraction angle, respectively. B, B_mea_ and b_ins_ are the breadths of intrinsic diffraction profile, the test sample diffraction integral profile and instrumental diffraction profile, respectively. UV–visible spectra were measured on UV-2450 UV spectrophotometer (Shimadzu Corporation, Japan). The range of the scanning wavelength was 200–800 nm. The BET specific surface area was measured by BELSOROP-MINI II (Japan) adsorption instrument and pore size distribution was analyzed by Barrett–Joyner–Halenda (BJH) method.

GC–MS (Agilent 7890A-5795C, America) was used for the component’s analysis. The instrument was equipped with a DB-5 capillary column (length of 30 m, 0.25 mm i.d., 0.25 mm d.f.). The injector and MS transmission line temperatures were 250 and 310 °C, respectively. The oven temperature initiated at 40 °C (hold for 5 min), and then increased at 5 °C min^−1^ to 290 °C (hold for 2 min). The electron energy was set at 70 eV and the ion source temperature was 230 °C. The standard spectra in GC–MS database was used to identify the chemical constituents in wastewater. In the present study, refractory acrylonitrile wastewater was obtained from an acrylonitrile manufacturing plant. The acrylonitrile wastewater was pretreated through adsorption with microporous zeolite, HZSM-5 before photocatalytic degradation, COD_Cr_ decreased from 582.4 to 88.36 mgL^−1^, and after that there were no change in the values. The organic pollutants in the wastewater after adsorption were detected and the results were listed in Table 1.

**Table 1:** The main organic pollutants in the industrial acrylonitrile wastewater after adsorption by HZSM-5.

S. no	Primary pollutants	Structural formula	Concentration (mgL^−1^)	
			Raw water	HZSM-5
1	Acetonitrile		10.45	2.24
2	Acrylonitrile		23.10	4.65
3	Acetone cyanohydrin		420.98	54.35
4	Toluene		10.56	3.14
5	Pyrazole		8.37	5.83
6	Phenanthrene		6.45	2.79

### Photocatalytic activities for acrylonitrile simulated wastewater

The photocatalytic activities of the photocatalyst used for acrylonitrile simulated wastewater were evaluated in a photocatalytic reaction system^20^. The quartz glass reactor was sealed after 180 mL of acrylonitrile wastewater (10 mgL^−1^) and 300 mg of the catalyst was placed in it. The mixture was magnetically stirred in the dark until the adsorption equilibrium attained. Then, the spherical Xenon short arc lamp (AHD350, 350 W) was turned on for 12 min. In the process of illumination, the reaction solution of 1 mL was taken out at the interval of 2 min, and after that photocatalyst was filtered out through a filter film of 0.45 μm, acrylonitrile concentration was measured by HP-LC with LC-2030 UV detector and Sunfire TM C18 column. The wavelength of detector was 210 nm and the mobile phase volume ratio of methanol to water was 3:7. Triplicate samples from each batch were taken for the tests.

### The ion effects on photocatalytic activities of acrylonitrile simulated wastewater

The ion effects were examined by following procedure: appropriate amount of sodium sulfate and sodium chloride were added to 180 mL of solution to obtain 61.8 mgL^−1^ of sulfate radical and 22 mgL^−1^ of chloride ion consistent with Table 2. The ion solution containing acrylonitrile was used for the comparative trial.

**Table 2:** Concentration of inorganic ions, BOD and COD_Cr_ in acrylonitrile raw wastewater and the wastewater after adsorption by HZSM-5.

Inorganic ions/COD_Cr_/BOD	Inorganic ion concentrations (mgL^−1^)	
	Raw water	HZSM-5
Phosphate	0.06	0.03
Nitrate	1.38	1.06
Sulphate	189	61.8
Chloride ion	44	22
Fluorine ion	0.17	0.16
Calcium ion	2.92	1.13
Ferric ion	0.17	0.055
Cyanide	0.017	0.011
COD_Cr_	582.4	88.36
BOD	1.15	2.24

### Activity evaluation by COD_Cr_ and TOC measurements for acrylonitrile wastewater after adsorbed by HZSM-5

The catalyst contents and reaction device were used as the same as that in simulated wastewater. After adsorption equilibrium, the spherical Xenon lamp was turned on for 14 h. At given intervals of illumination, 4 mL of reaction solution was taken out and was filtered out through a filter film of 0.45 μm (all detection procedures in this study were performed according to or as per China national standard except for special mention). COD_Cr_ values were detected with a Fast COD Detection Instrument (LH-5B-3B(V8)). TOC concentrations of the samples were measured via a Shimadzu TOC analyzer (TOC-L CPN, Japan). Concentration of inorganic ions, BOD and COD_Cr_ in acrylonitrile raw wastewater, the wastewater after adsorption measured according to national standard methods were listed in Table 2.

## Results

### Effect of different ions on the activity and light adsorption performance

**Figure 1.**
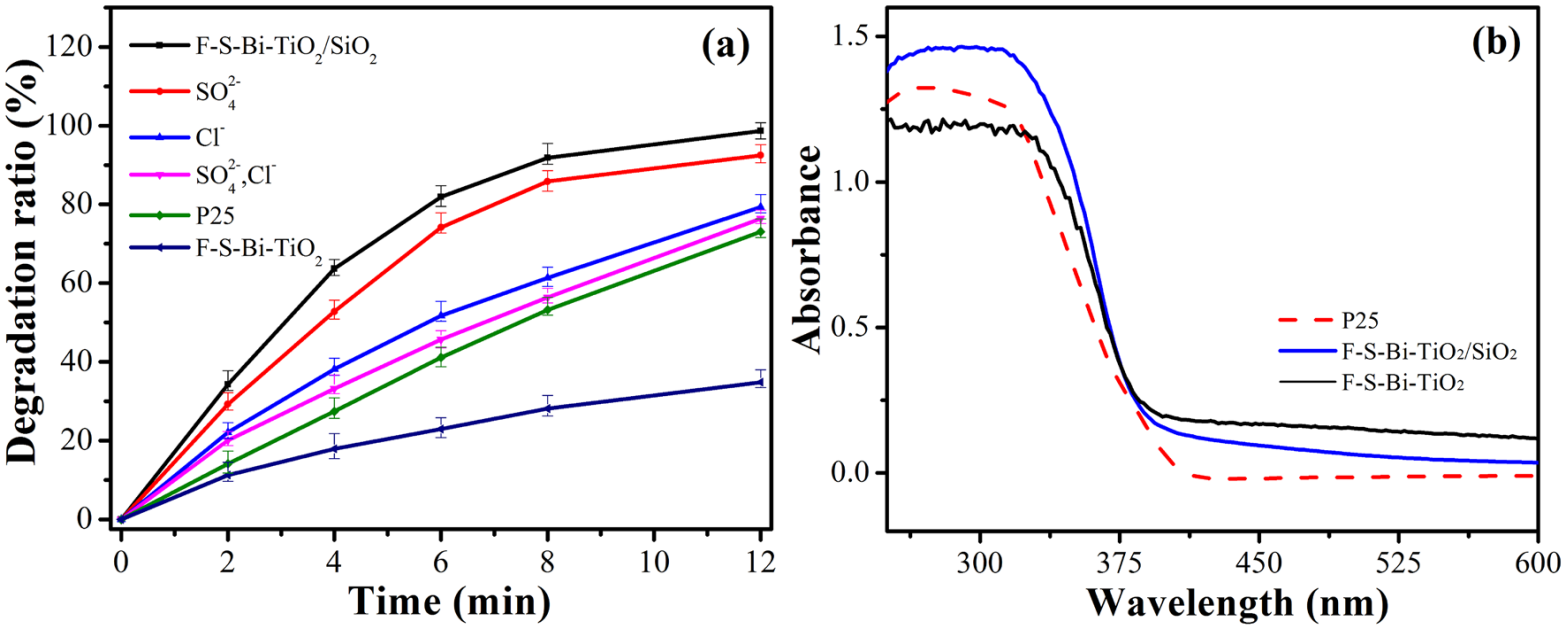
(**a**) Photocatalytic activities of acrylonitrile simulated wastewater degradation of SO_4_^2−^, Cl^−^, TiO_2_–P25, F–S–Bi–TiO_2_, and F–S–Bi–TiO_2_/SiO_2_; (**b**) UV–visible spectra of TiO_2_–P25, F–S–Bi–TiO_2_/SiO_2_ and F–S–Bi–TiO_2_.

Figure 1a shows the photocatalytic performances of the prepared samples for acrylonitrile simulated wastewater under the effect of spherical Xenon short arc lamp. The degradation ratio of F–S–Bi–TiO_2_/SiO_2_ catalyst after 4 min was 63%, which was much higher than TiO_2_–P25 and F–S–Bi–TiO_2_.

In order to demonstrate the interference of inorganic ions, a certain amounts of sodium sulfate (61.8 mgL^−1^ sulfate radical) and sodium chloride (22 mgL^−1^ chloride ion) were added into acrylonitrile simulated wastewater and the degradation was carried out by the catalyst under the same condition mentioned above. These results revealed that free sulfate radical, chloride ions, or sodium ions inhibited the degradation progress. This was similar with our previous studies which illustrated that, certain inhibition effect by free sulfate radical on the degradation process^22^.

Figure 1b shows the UV–Visible spectra of TiO_2_ P-25 (Degussa), F–S–Bi–TiO_2_/SiO_2_ and F–S–Bi–TiO_2_ catalysts. The absorption of F–S–Bi–TiO_2_/SiO_2_ catalyst was increased in the UV–visible region at 300–600 nm as compared to TiO_2_ P-25. However, the UV–visible spectrum of F–S–Bi–TiO_2_ became flat below 335 nm, showing weakest absorbance. Although, F–S–Bi–TiO_2_/SiO_2_ exhibited lower absorption ability towards visible region (400–600 nm) than F–S–Bi–TiO_2_, showing the higher degradation ratio. The higher degradation ratio was attributed to the better dispersion of SiO_2_, because in F–S–Bi–TiO_2_/SiO_2_ photocatalyst TiO_2_ was well dispersed, reducing the agglomeration and enhancing the absorption in the UV region^22^. However, both the photocatalysts have the same weight but F–S–Bi–TiO_2_ contains more S elements and absorbed more visible light. The Photocatalytic reaction mainly depends upon the UV light.

### XRD analysis

In order to know the crystal structure of the prepared catalyst, XRD patterns of F–S–Bi–TiO_2_/SiO_2_ and F–S–Bi–TiO_2_ calcinated at 450 °C were recorded as shown in Fig. S2. The crystallite size of the catalysts was calculated with the most predominant peak of the anatase face (101) with the help of Scherrer equation (Table 3). It was clearly revealed from Table 3 that the addition of SiO_2_ as a dispersant agent decreases the average crystal size, and increases the surface area of the photocatalyst.

**Table 3:** The average crystal size, specific surface area and average pore diameter of F–S–Bi–TiO_2_ and F–S–Bi–TiO_2_/SiO_2_ samples.

Components	F–S–Bi–TiO_2_	F–S–Bi–TiO_2_/SiO_2_
Average crystal size (nm)	40.4	13.9
Specific surface area (m^2^/g)	45.9	194.3
Average pore diameter (nm)	26.0	14.1

### TEM and EDS analysis

**Figure 2.**
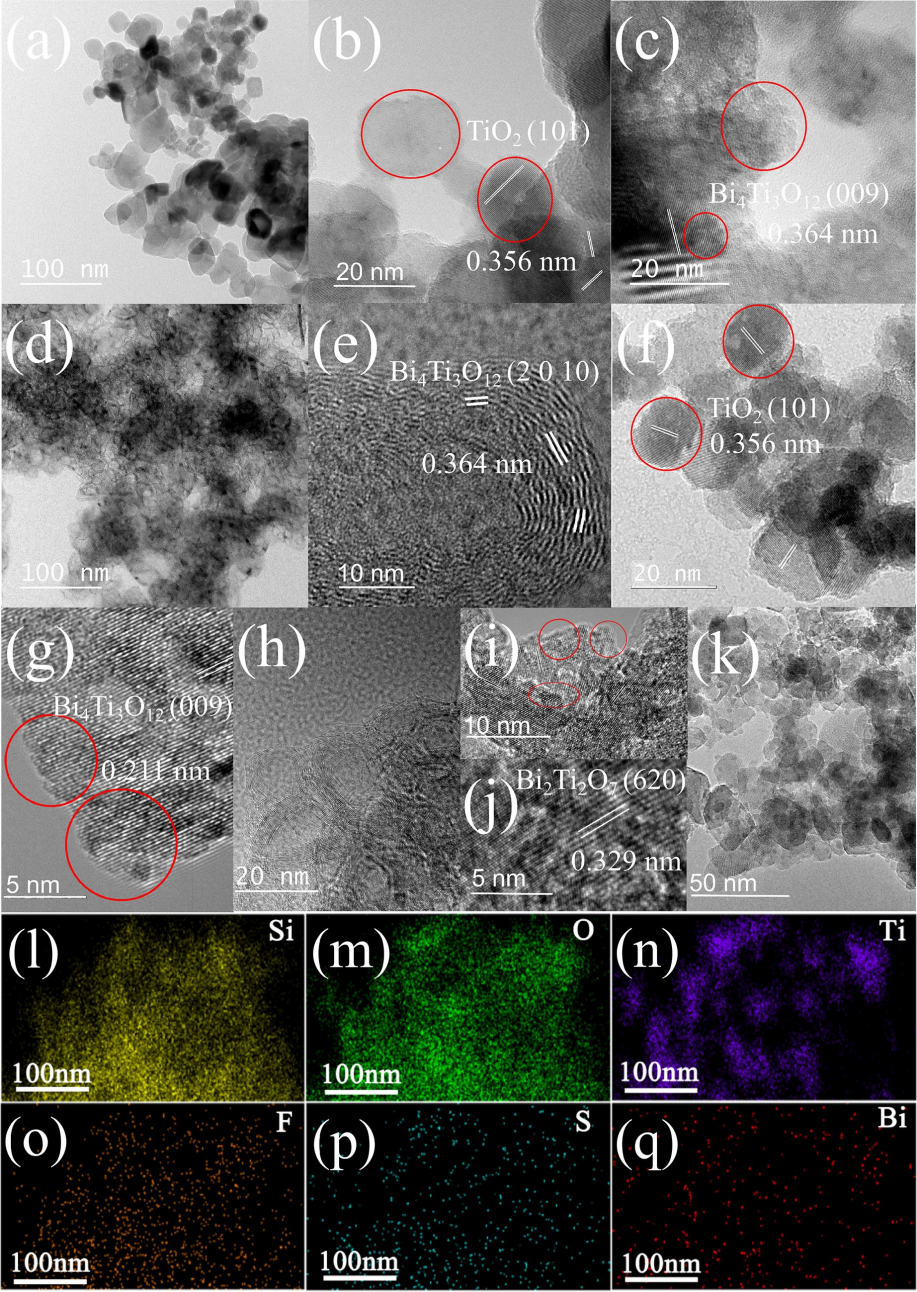
TEM images of, (**a**–**c**) F–S–Bi–TiO_2_ and (**d**–**k**) F–S–Bi–TiO_2_/SiO_2_ samples. (**l**–**q**) EDS mapping of Si, O, Ti, F, S and Bi elements of S–Bi–F–TiO_2_/SiO_2_ samples.

The TEM images of as prepared photocatalyst without SiO_2_ were shown in Fig. 2a–c. From Fig. 2a, we observed the crystal with grains size of 11–60 nm, and they might be formed in different aggregated stages. Aggregation of *ca*. 4 nm of particles were produced on the rough end faces, where borderline disappeared in the interior of the large piece crystal (Fig. 2b). The other type gave out indistinct borderline in Fig. 2c. The crystallite faces of TiO_2_ (d = 0.356 nm, (101)) and Bi_4_Ti_3_O_12_ (d = 0.364 nm, (009)) were exhibited in Fig. 2b,c. Package morphology has been identified clearly by TEM images (Fig. 2d). The atoms are arranged on irregular crystal planes of several nanometers of out layer films. The core surface was most rough (Fig. 2e). The package morphology was formed after the addition of SiO_2_. The black parts in the figure represented the pores in SiO_2_. The other general crystal configurations look like bulk type of TiO_2_ and Bi_4_Ti_3_O_12_ (Fig. 2f,g). As comparison to Fig. 2b, TiO_2_ crystal with SiO_2_ was also constituted by smaller particles of 2–4 nm with identical crystal face (Fig. 2f). Bi_4_Ti_3_O_12_ (d = 0.211 nm, (2010)) crystal with borderline mark aggregated into a large crystal and also possessed identical crystal face (Fig. 2g). The explanation was that crystal growth might influence each other in same aggregate through the interface, connected among gel particles to form relatively larger identical crystal face, like as biomimetic crystallization. Based on the same principle of interaction of end face atoms, different crystal faces were developed at starting on an end face on the basis of total lowest-energy rule of the system (Fig. 2i). Bi_2_Ti_2_O_7_ in Fig. 2j was regarded as the intermediate phase of Bi_4_Ti_3_O_12_ formation^25^. A large number of bumps were also formed on the rough surfaces (Fig. 2k). The packages were formed by silk ribbon-like film twining (Fig. 2h). This film could be consisting of long identical crystal face and generated from stirring drawing. Relatively large mass of SiO_2_ promoted drawing in late stage of gelation. The EDS images in Fig. 2 show that Si, O, Ti, F, S and Bi elements were evenly distributed on the surface of S–Bi–F–TiO_2_/SiO_2_ catalyst^26^, which confirmed the conjecture that the elements were not detected in XRD.

**Figure 3.**
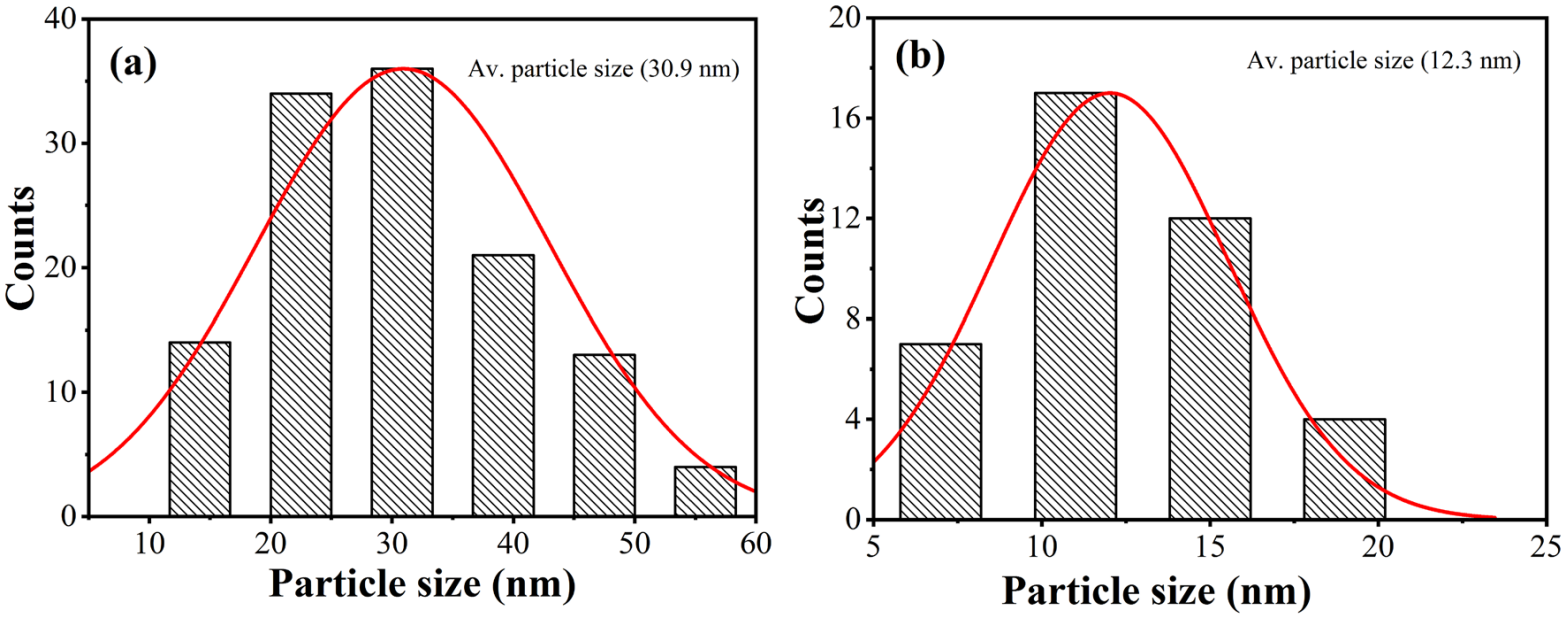
Particle size distributions of (**a**) F–S–Bi–TiO_2_ and (**b**) F–S–Bi–TiO_2_/SiO_2_ samples.

The particle size distribution of the catalyst was calculated with NIH software for all particles in random region and was shown in Fig. 3. The catalyst without SiO_2_ showed a wide particle size distribution. The average diameter was 30.9 nm, which was smaller than crystal grain size (40.4 nm) as calculated by XRD (Table 3). Since the borderline was not identified by the program, we measured the diameters of particles artificially and calculated particle size distribution of the catalyst with SiO_2_ on the basis of the same rule. The result was shown in Fig. 3b. It has been found that the average diameter of the particles is 12.3 nm, which was in agreement with the XRD result (13.9 nm). Compared to the catalyst without SiO_2_, nanoparticles sizes are apparently different. Nanoparticles sizes of the major parts were above *ca,* 20 nm for the catalyst without SiO_2_, contrarily, they were below 16 nm for the catalyst with SiO_2_ and the major parts belonged to ultrafine nanoparticles^27^.

### Nitrogen physical adsorption

**Figure 4.**
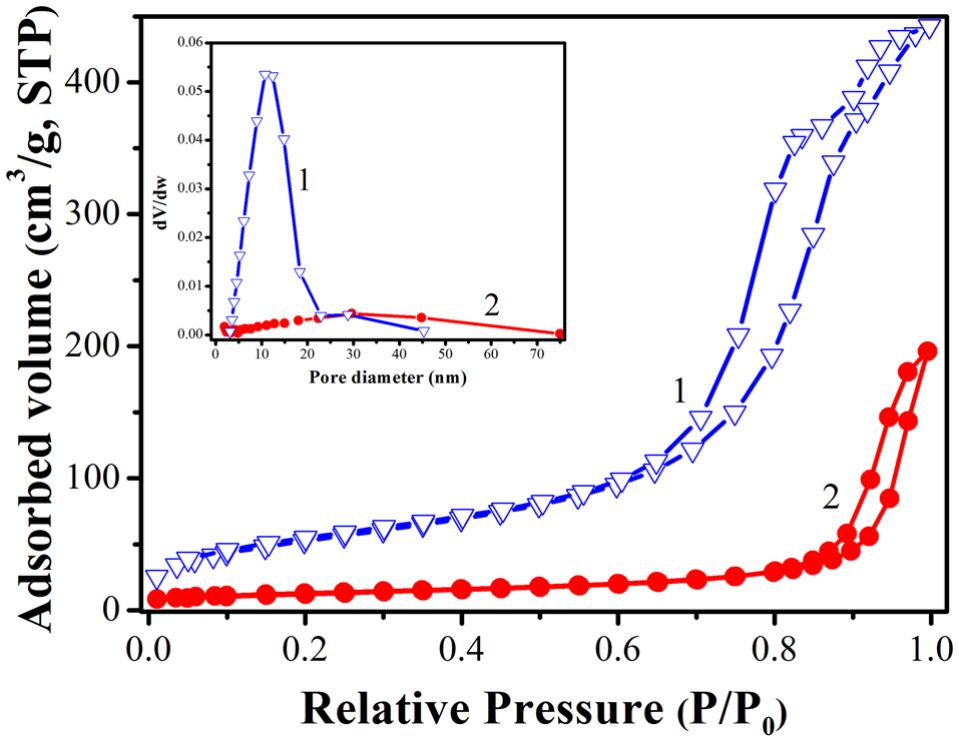
Nitrogen adsorption and desorption isotherms of (**1**) F–S–Bi–TiO_2_/SiO_2_ and (**2**) F–S–Bi–TiO_2_. Inset shows BJH pore size distributions of the corresponding samples.

Figure 4 represents the nitrogen adsorption and desorption isotherms of F–S–Bi–TiO_2_/SiO_2_ and F–S–Bi–TiO_2_ calcined at 450 °C. Both F–S–Bi–TiO_2_/SiO_2_ and F–S–Bi–TiO_2_ display type IV isotherm and H_2_ hysteresis, which indicate the presence of mesoporous materials.

Moreover, the F–S–Bi–TiO_2_/SiO_2_ sample has the similar pore structure with F–S–Bi–TiO_2_. The inset in Fig. 4 shows the plot for the pore size distribution determined by Barrett–Joyner–Halenda (BJH) method from the adsorption branch of the isotherm. The average pore diameters and total pore volume of F–S–Bi–TiO_2_ were 26.0 nm, 0.30 cm^3^/g and for F–S–Bi–TiO_2_/SiO_2_ are 14.1 nm, 0.67 cm^3^/g. Both of them exhibit mesopore rich structure. However, at all pressure region, the adsorption amount of N_2_ on F–S–Bi–TiO_2_/SiO_2_ was higher than that of F–S–Bi–TiO_2_, which indicates that a lot of relatively small mesoporous on the surface of F–S–Bi–TiO_2_/SiO_2_ were formed under the action of SiO_2_. Therefore, the specific surface area of F–S–Bi–TiO_2_/SiO_2_ (194.3 m^2^/g) was higher than that of F–S–Bi–TiO_2_ (45.9 m^2^/g). And, greater adsorption capacity will lead to greater degradation rate, which was consistent with the experimental results.

### XPS analysis

**Figure 5.**
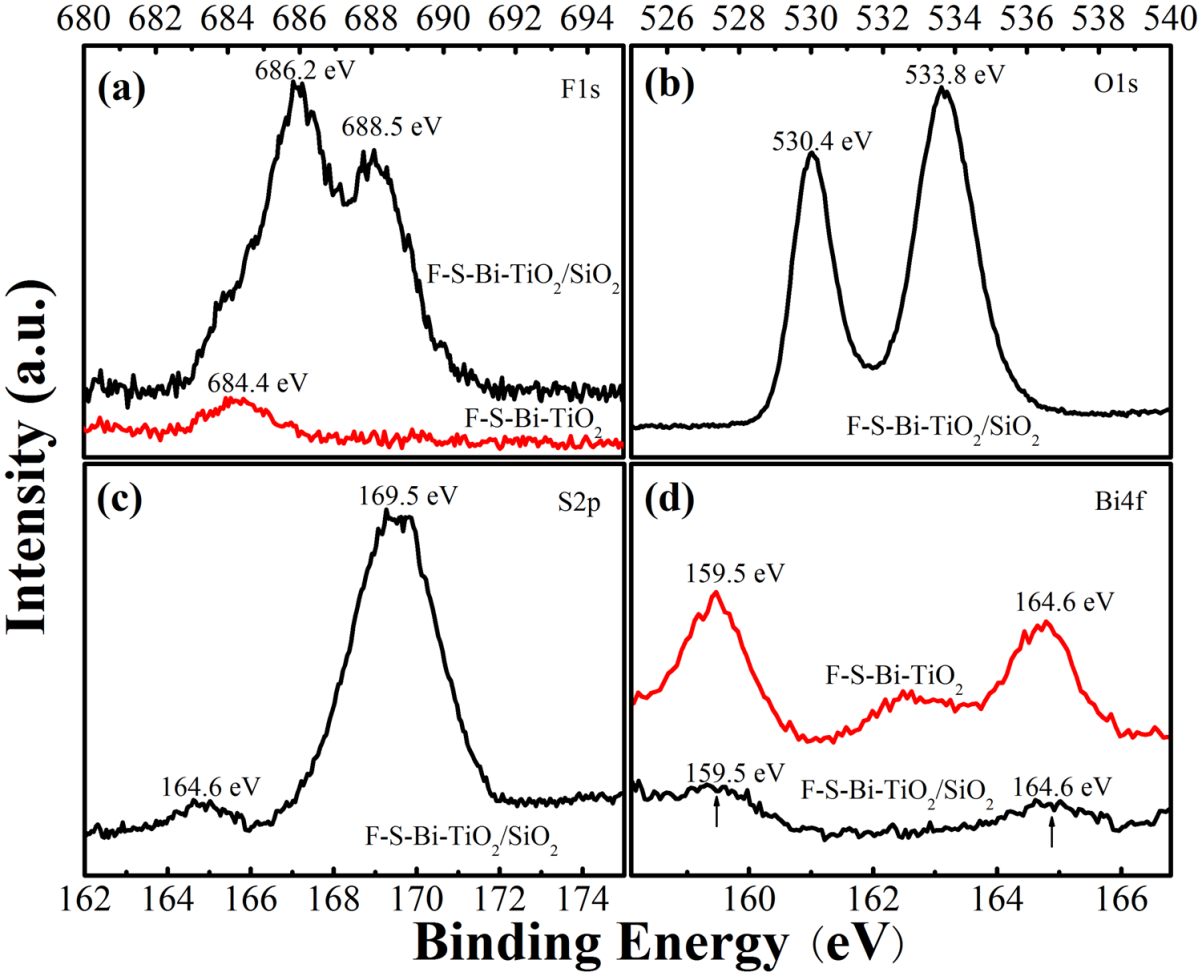
XPS spectra of F–S–Bi–TiO_2_ and F–S–Bi–TiO_2_/SiO_2_ samples: (**a**) F 1*s* spectra; (**b**) O 1*s* spectrum; (**c**) S 2*p* spectrum; (**d**) Bi 4*f* spectra.

XPS spectra of F–S–Bi–TiO_2_/SiO_2_ and F–S–Bi–TiO_2_ were shown in Fig. 5. Since fluorine doping converted Ti^4+^ state to Ti^3+^, and these Ti^3+^ state was related to oxygen vacancies^28^, the content of F changes in both types of the catalysts were investigated. In Fig. 5a the F 1*s* spectrum of F–S–Bi–TiO_2_ shows only one peak centered at binding energy 684.4 eV (represented by red color), indicated only surface fluoride species were present in F–S–Bi–TiO_2_. However, in case of F–S–Bi–TiO_2_/SiO_2_, the F 1*s* XPS spectra shows two peaks centered at binding energy 688.5 and 686.2 eV, respectively (represented by black color). The peak at binding energy 688.5 eV was attributed to the doped F into the substituted sites of TiO_2_ lattice and produced mixed oxide structure of O–Ti–F^18,29,30^. The lower binding energy, centered at 686.2 eV was attributed to the surface fluoride species adsorbed on the surface of TiO_2_^30^. This result shows that silica plays a very important role in stabilizing the dispersion of F ions. Next, the O 1*s* spectrum of F–S–Bi–TiO_2_/SiO_2_ catalyst was shown in Fig. 5b. The peak at binding energy 533.8 eV was attributed to oxygen present in surface hydroxyl species^29^, while the peak at binding energy 530.4 eV ascribed to lattice oxygen of TiO_2_^16^. Next, the S 2*p* spectra of the catalyst was shown in Fig. 5c. A well symmetrical S 2*p* peak at binding energy 169.5 eV was observed, corresponding to S^6+^ state of SO_4_^2−^ species^31,32^. The binding energy at 164.6 eV was attributed to elemental sulfur^33^, which was overlapped with Bi 4*f* XPS spectra. Hence, the SO_4_^2−^ species were mainly adsorbed on the catalyst surface, which improved surface acid strength and favored to the adsorption and degradation of the pollutants^22,34^. The Bi 4*f* XPS spectrum was shown in Fig. 5d. The peak at binding energy 159.5 eV was attributed to Bi 4*f*_7/2_, while the peak at binding energy 164.7 eV belonged to Bi 4*f*_5/2_. These values of binding energies were higher than the binding energy of Bi^3+^, indicating that bismuth existed in Bi^3+δ^ state and formed Bi–O–Ti bond^16^. The intensities of Bi 4*f* peaks of F–S–Bi–TiO_2_/SiO_2_ were decreased as compared to F–S–Bi–TiO_2_, which indicate that dispersion function of silica was selective, that was only favorable for the substitution of F for the lattice oxygen but not for the formation Bi oxides. The weight percentages of the elements in the F–S–Bi–TiO_2_/SiO_2_ and F–S–Bi–TiO_2_ were shown in Table S2.

### Photocatalytic purification of industrial acrylonitrile wastewater

**Figure 6.**
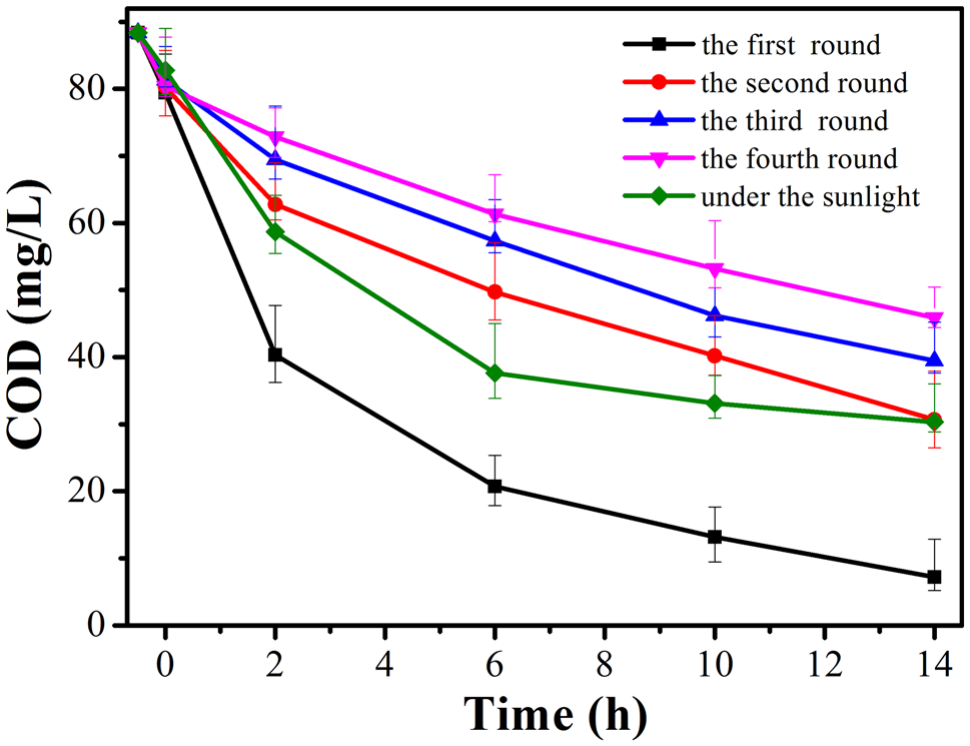
COD_Cr_ change as a function of time in wastewater of recycle reactions using F–S–Bi–TiO_2_/SiO_2_ catalyst under natural sunlight irradiation.

On the basis of above analysis (Tables 1 and 2) we found that, there were a lot of inorganic and organic substances in the industrial acrylonitrile wastewater and these substances inhibited the catalyst activity (Fig. 1a). Specially, low ratio of BOD to COD_Cr_ (Table 2) identified that biochemical treatment could not be used. On the other hand, since there was limitation in amount of adsorption (Fig S1), the use of adsorption to treat was also hardly to reach regulation discharge. Hence, photocatalytic degradation of industrial acrylonitrile wastewater was one of most promising technique. A stable light source was required to keep experimental repeatability as mentioned in Table S1. Figure 6 shows the change in the value of COD_Cr_ as a function of illumination time under simulated sunlight. It was found that COD_Cr_ value decreased from 88.36 to 7.20 mgL^−1^ and TOC concentration decreased from 39.45 to 2.57 mgL^−1^ for 14 h irradiation. F–S–Bi–TiO_2_/SiO_2_ catalyst was recycled through filtration after reaction and the recovery ratio was more than 95%. The photocatalytic reaction performance of recycled F–S–Bi–TiO_2_/SiO_2_ was demonstrated through repeated rounds under the same reaction conditions. The value of COD_Cr_ was 30.67 mgL^−1^ after 14 h of illumination at the second round. After photocatalysis, the value of COD_Cr_ was 45.86 mgL^−1^ at the fourth round.

Figure 6 shows the photocatalytic activity of fresh F–S–Bi–TiO_2_/SiO_2_ catalyst for the degradation of pollutants in industrial acrylonitrile wastewater under natural sunlight irradiation. The value of COD_Cr_ decreased to 39 mgL^−1^ after 6 h irradiation, which has satisfied China national discharge standards.

## Discussion

The actual textile dyeing wastewater was effectively oxidized by TiO_2_ under UV radiation. The degradation percentage of the dye and COD_Cr_ were 98.50% and 91.50%, respectively^11^. Dai et al.^25^ investigated the adsorption purification of acrylonitrile production wastewater by a microporous zeolite, CS-Z1 and a visible light-driven Ti–*β*–Bi_2_O_3_ photocatalyst. Nanoporous Ti–*β*–Bi_2_O_3_ was prepared via a solvothermal synthesis method in laboratory. Dai et al. was mainly interested in the theory, but not in application. They have not considered the inorganic ions effects. In this paper, we used industrial acrylonitrile wastewater as testing samples. The industrial acrylonitrile wastewater was pretreated by microporous zeolite, HZSM-5. After the treatment, the wastewater contained some inorganic and organic matter, as shown in Tables 1 and 2. Some of them were same or different to the pollutants reported by Dai et al. Table 2 only gives out a part of interfering inorganic ions And the existence of these ions could slow down the reaction rate (Fig. 1a). Our results showed that the prepared catalyst possesses certain anti-interfering ability. From the application point of view, we have used simple SiO_2_ dispersing sol–gel for the synthesis of highly active ultrafine nanoparticle catalyst with certain anti-ion interfering function and for the first time we successfully degraded industrial acrylonitrile wastewater in only 6 h under natural sunlight. This is the most significant findings for the photocatalysis application in environment.

For most of the pollutants, close to zero discharge is a final goal that people pursue in environment protection. Sixto et al*.*^10^ have demonstrated that photocatalytic purification of phenol containing wastewater by TiO_2_-P25 using sacrificial agent under ultraviolet part of sunlight. In this study, there was not any sacrificial agent, however, we still realized that the values of COD_Cr_ and TOC near zero discharge. These results show the potential of as prepared photocatalyst in near zero discharge and high TOC removal efficiency of wastewater treatment.

In our previous study, we found that the reaction rate of the catalyst with SiO_2_ was several times faster than without SiO_2_. The later exhibited some general properties of a large bulk aggregate. On the other hand, there were a majority of ultrafine nanoparticles with acidic sites on the catalyst with SiO_2_^20^. A large number of bumps increased the quantum size effect and UV absorption at 270–380 nm, which should raise activity. Several nanometers package of irregular crystal films have promotion effect on light absorption. Photon were generated by light which can go through several nanometers of irregular Bi_4_Ti_3_O_12_ out layer film and get into TiO_2_ (Fig. 2e), causes scattering at the rough interface of the both phases. Apparently, it should be favorable to light absorption and to promote the catalytic activity (Fig. 6). The bumps were different with films of packages in morphology, but we think that were consistent in functions, because their sizes were in approximate ranges of several nanometers (Fig. 2e, g). Apparently, there were much more F^-^ ion in the lattice, which could produce holes^19^ and also contribute the high activity of the catalyst with silica dispersion.

## Conclusion

We built up a most simple and low cost approach for the preparation of ultrafine nanocatalyst of F, S and Bi doped TiO_2_ with SiO_2_ dispersing sol–gel particles and successively degraded the organic pollutants in acrylonitrile industrial wastewater to reach national discharge standard under 6 h of natural sunlight irradiation. In the prepared photocatalyst the doping of F, S and Bi causes the enhanced absorbance in the visible region. The results of photocatalytic activity evaluation demonstrated that COD_Cr_ value reached to discharge standard still after four recycle uses under the simulated sunlight irradiation, and to near zero discharge for the fresh photocatalyst. These results exhibited effectiveness and potential of our photocatalyst for the treatment of complicated and refractory industrial wastewater. The XPS and EDS analysis implied that S and Bi were doped successfully. The photocatalyst size distribution has been identified by visible nano-aggregates, constructed with 2–4 nm of finer gel particles. It indicated the function of SiO_2_ dispersing to form ultrafine nanoparticles. The nano-aggregates might form an identical lattice faces or different faces when gel particles crystallized. TEM results revealed that, the bump’s numbers may also be responsible for the increase in the light adsorption and photocatalytic activity. The identical face might originate from silk ribbon film of package. The crystals of several nanometers of out layer films and rough core surfaces of packages also increased the light absorption and enhance their photocatalytic activity.

## Supplementary information

Supplementary Information.
