# Supplementary material for: Photocatalytic degradation of industrial acrylonitrile wastewater by F–S–Bi–TiO2 catalyst of ultrafine nanoparticles dispersed with SiO2 under natural sunlight
Source: Sci Rep. 2020 Jul 23;10:12379. doi: 10.1038/s41598-020-69012-z (PMC7378175; doi:10.1038/s41598-020-69012-z)
Supplement: Supplementary file 1 — Supplementary Information. [file 41598_2020_69012_MOESM1_ESM.pdf]

Appendix A. Supplementary data

Photocatalytic degradation of industrial acrylonitrile wastewater by F-S-Bi-TiO_2 catalyst of ultrafine nanoparticles dispersed with SiO_2 under natural sunlight

Feng (SY) Ouyang[a, b]*, Hanliang Li[b]†, Zhengya Gong[b], Dandan Pang[c]†, Lu Qiu[b, d]*, Yun Wang[b], Fangwei Dai[b], Gang Cao[e] & Bandna Bharti[b]

[a] *State Key Laboratory of Urban Water Resource and Environment, Harbin Institute of Technology, Harbin 150090, P.R. China*

[b] *School of Civil and Environmental Engineering, Harbin Institute of Technology, Shenzhen 518055, PR China.*

[c] *Henan University of Urban Construction, Pingdingshan 467036, PR China.*

[d] *Tonson Tech Automation Equipment CO., Ltd, Shenzhen 518100, PR China.*

[e] *Shenzhen Key Laboratory of Organic Pollution Prevention and Control, Harbin Institute of Technology, PR China.*

†Co-first author; *Corresponding author: e-mail address: ouyangfh@hit.edu.cn; 329967681@qq.com

This Supplementary data contains the detailed information as follow, Table S1–S2, and Fig. S1–S4.

Preparation of photocatalysts. Firstly, solution A was made up according to our previous method[1]. CH_4N_2S (24 mg), $Bi(NO_3)_3 \cdot 5H_2O$ (49 mg) and HF (50%, 0.7 ml) were dissolved into the mixed solution of anhydrous alcohol (21 mL), acetic acid (4.2 mL) and deionized water (1.05 mL) and then stirred to form solution B. Solution B was added dropwise into solution A with vigorous stirring. After that a certain amount of silica gel (100~200 mesh) was added into the sol with agitation until gelatinous solution was formed. After aging at room temperature for 10 h, the solid was dried at 80 ^{o}C for several hours, and calcined at 450 ^{o}C in air for 2 h. Thus, F, S and Bi doped TiO_2/SiO_2 powders were obtained.

Pretreatment of acrylonitrile industrial wastewater adsorbed with microporous zeolite (HZSM-5). For efficient photocatalytic degradation, the acrylonitrile industrail wastewater was pretreated. The adsorption was conducted by the following procedure: firstly, 8.5 g zeolite

(HZSM-5) was calcined at 450 °C in air for 5 hours and activated. Then, it was added to the acrylonitrile wastewater and shaken for different times, finally achieved equilibrium after 24 hours of adsorption (Fig. S1). The pretreated wastewater was used for the photocatalytic degradation.

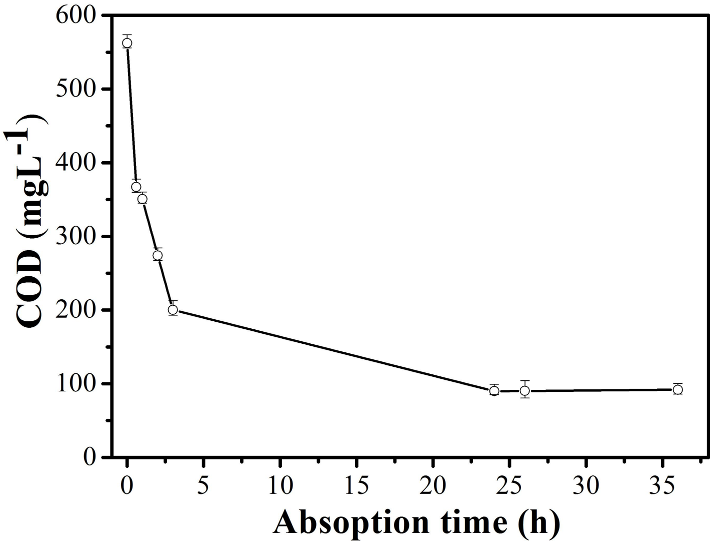

Figure. S1 The change in COD values with adsorption times by HZSM-5.

Biochemical oxygen demand (BOD) measurements. Preparation of dilution water: 160 mL of Dasha River water, that was aerated fully for 2 h was added into 3200 mL of deionized water and keep standing them for 1 hour to prevent oxygen saturation. The phosphate buffer solution, magnesium sulfate solution, calcium oxide solution and ferric chloride solution were added into the water by 1 mLL^{-1} respectively.

Experimental process: 150 mL of raw water, water after adsorption or water after reaction were put in the dissolved oxygen bottles with a water sealing device, adding dilution water, measuring dissolved oxygen. And an incubator was used for temperature and humidity control. Finally, dissolved oxygen values were determined by the following equation:

$$\rho = [\ (\rho_1 - \rho_2) - (\rho_3 - \rho_4) f_1\ ] / f_2 \qquad \text{(S1)}$$

Where ρ is five days biochemical oxygen demand; ρ_1 is the dissolved oxygen concentration in inoculated dilution water sample before culture mgL^{-1}; ρ_2 is the dissolved oxygen concentration in inoculated dilution water sample after culture mgL^{-1}; ρ_3 is the dissolved oxygen concentration in blank water sample before culture mg·L^{-1}; ρ_4 is the dissolved oxygen concentration in blank water sample after culture mg·L^{-1}; f_2 is the ratio of inoculated dilution water to dilution water in nutrient solution, and f_1 is the raw sample proportion in nutrient solution.

Table S1

Irradiation intensity of natural sunlight in August 20, 2017 and the spherical Xenon short arc lamp.

Time (h)	Sunlight (mW/cm^2)	Xe lamp (mW/cm^2)
6:00	10.40	126.6
7:00	27.60	126.6
8:00	52.34	126.6
9:00	66.80	126.6
10:00	69.00	126.6
11:00	70.00	126.6
12:00	71.57	126.6
13:00	77.76	126.6
14:00	69.28	126.6
15:00	62.00	126.6
16:00	39.97	126.6
17:00	32.54	126.6
18:00	28.14	126.6

19:00	15.60	126.6
20:00	0	126.6

According to Table S1, there exist sunlight irradiation at 6-20 o'clock on this day. And the sunlight irradiation is mainly concentrated at 8-12 o'clock and 12-16 o'clock, so we choose a 4 h interval. In the initial stage of the photocatalytic reaction, the reaction rate is fast, and a shorter time interval is required (2 h, 6-8 o'clock), and 16-20 o'clock is the last interval.

Table S2

The weight percentages of the elements present in F-S-Bi-TiO_2 and F-S-Bi-TiO_2/SiO_2 photocatalyst.

F-S-Bi-TiO_2 Elements	Wt. percentage	F-S-Bi-TiO_2/SiO_2 Elements	Wt. percentage
Bi	4.235	Bi	0.2266
S	1.371	S	1.512
Ti	54.57	Ti	20.71
O	38.98	O	56.65
F	0.8540	F	11.13
		C	9.773

XRD analysis. The prepared samples had been characterized by peaks at 2θ= 25.36°, 37.91°, 48.15°, 54.05°, 55.20°, 62.86°, 68.97°, 70.47°, 75.30°, corresponding to (101), (004), (200), (105), (211), (204), (116), (220) and (225) crystal faces of anatase phase of TiO_2 (PDF#21-1272), respectively. There were no diffraction peaks of Bi sulfides or Bi_2O_3 due to its low content, and no other crystal phases were detected by XRD.

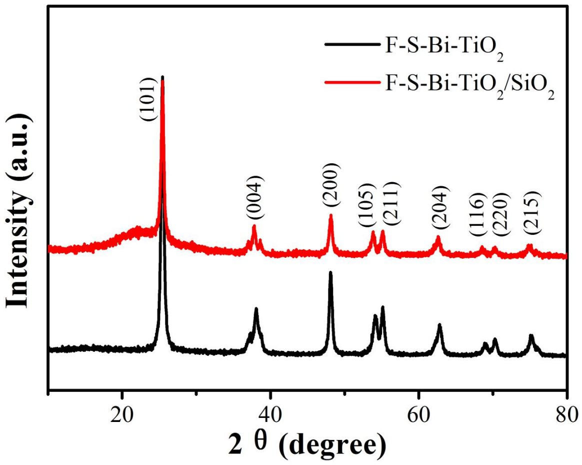

Figure. S2 XRD patterns of F-S-Bi-TiO_2 and F-S-Bi-TiO_2/SiO_2 samples.

Photocatalytic Mechanism of S-Bi-F-TiO_2/SiO_2 photocatalyst. We have discussed the band-structure in previous article[1]. As shown in Fig. S3, F doping led to the creation of surface oxygen vacancies and the increase of Ti^{3+} ion contents. S doping increases acid strength and enhance absorption in visible region. Bi doping mainly inhibits the recombination of photogenerated electron-hole pairs.

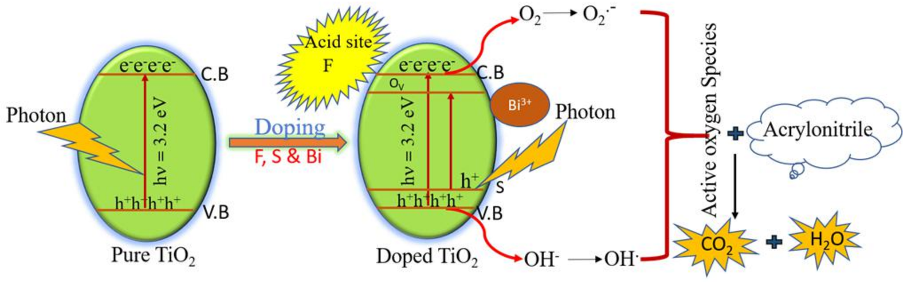

Figure. S3 Proposed mechanism involved in the photocatalytic degradation of acrylonitrile by F-S-Bi-TiO_2/SiO_2.

Main active species[1]. During the photocatalytic reaction, the active species of oxidation mainly included hydroxyl radicals ($\cdot OH$), hole (h^+) and superoxide anion ($O_2^{\cdot -}$). Sacrificial agents were added to distinguish the activity of oxidative species during the photocatalytic degradation of acrylonitrile, including IPA for $\cdot OH$, EDTA for h^+ and BQ for $O_2^{\cdot -}$. Fig. S4 demonstrates the photocatalytic degradation of acrylonitrile by S-Bi-F-TiO_2/SiO_2 under simulated sunlight

through adding different sacrificial agents. Results reveal ·OH radical is the highest reactive oxygen species which could extensively oxidize acrylonitrile.

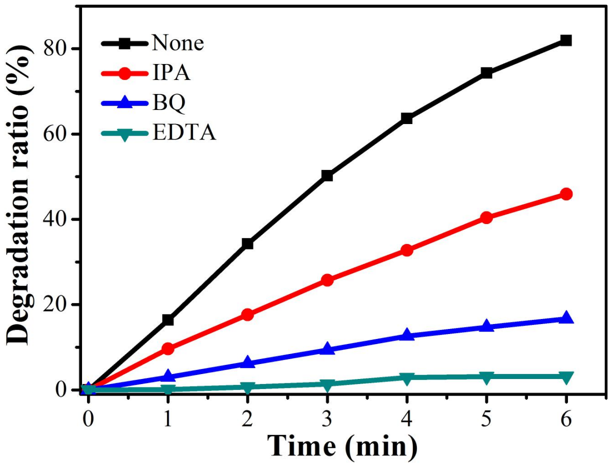

Figure. S4 Effects of different sacrificial agents on the photodegradation activity of acrylonitrile with F-S-Bi-TiO_2/SiO_2 catalyst.

References:

1. Li, H. L. *et al*. Efficient photocatalytic degradation of acrylonitrile by Sulfur-Bismuth co-doped F-TiO_2/SiO_2 nanopowder. *Chemosphere.* **249**, 126135 (2020).
